# Hematopoietic cell-mediated dissemination of murine cytomegalovirus is regulated by NK cells and immune evasion

**DOI:** 10.1371/journal.ppat.1009255

**Published:** 2021-01-28

**Authors:** Shunchuan Zhang, Lauren E. Springer, Han-Zhi Rao, Renee G. Espinosa Trethewy, Lindsey M. Bishop, Meaghan H. Hancock, Finn Grey, Christopher M. Snyder

**Affiliations:** 1 Department of Microbiology and Immunology, Sidney Kimmel Medical College, Sidney Kimmel Cancer Center, Thomas Jefferson University, Philadelphia, Pennsylvania, United States of America; 2 Vaccine and Gene Therapy Institute, Oregon Health & Science University, Beaverton, Oregon, United States of America; 3 Division of Infection and Immunity, The Roslin Institute, University of Edinburgh, Easter Bush, Midlothian, United Kingdom; ETH Zurich, SWITZERLAND

## Abstract

Cytomegalovirus (CMV) causes clinically important diseases in immune compromised and immune immature individuals. Based largely on work in the mouse model of murine (M)CMV, there is a consensus that myeloid cells are important for disseminating CMV from the site of infection. In theory, such dissemination should expose CMV to cell-mediated immunity and thus necessitate evasion of T cells and NK cells. However, this hypothesis remains untested. We constructed a recombinant MCMV encoding target sites for the hematopoietic specific miRNA miR-142-3p in the essential viral gene IE3. This virus disseminated poorly to the salivary gland following intranasal or footpad infections but not following intraperitoneal infection in C57BL/6 mice, demonstrating that dissemination by hematopoietic cells is essential for specific routes of infection. Remarkably, depletion of NK cells or T cells restored dissemination of this virus in C57BL/6 mice after intranasal infection, while dissemination occurred normally in BALB/c mice, which lack strong NK cell control of MCMV. These data show that cell-mediated immunity is responsible for restricting MCMV to hematopoietic cell-mediated dissemination. Infected hematopoietic cells avoided cell-mediated immunity via three immune evasion genes that modulate class I MHC and NKG2D ligands (m04, m06 and m152). MCMV lacking these 3 genes spread poorly to the salivary gland unless NK cells were depleted, but also failed to replicate persistently in either the nasal mucosa or salivary gland unless CD8^+^ T cells were depleted. Surprisingly, CD8^+^ T cells primed after intranasal infection required CD4^+^ T cell help to expand and become functional. Together, our data suggest that MCMV can use both hematopoietic cell-dependent and -independent means of dissemination after intranasal infection and that cell mediated immune responses restrict dissemination to infected hematopoietic cells, which are protected from NK cells during dissemination by viral immune evasion. In contrast, viral replication within mucosal tissues depends on evasion of T cells.

## Author summary

Cytomegalovirus (CMV) is a common cause of disease in immune compromised individuals as well as a common cause of congenital infections leading to disease in newborns. The virus is thought to enter primarily via mucosal barrier tissues, such as the oral and nasal mucosa. However, it is not clear how the virus escapes these barrier tissues to reach distant sites. In this study, we used a mouse model of CMV infection. Our data illustrate a complex balance between the immune system and viral infection of “myeloid cells”, which are most commonly thought to carry the virus around the body after infection. In particular, our data suggest that robust immune responses at the site of infection force the virus to rely on myeloid cells to escape the site of infection. Moreover, viral genes designed to evade these immune responses were needed to protect the virus during and after its spread to distant sites. Together, this work sheds light on the mechanisms of immune control and viral survival during CMV infection of mucosal tissues and spread to distant sites of the body.

## Introduction

Cytomegalovirus (CMV), is the most common infectious cause of birth defects in the developed world, leading to hearing loss, vision impairment and cognitive/motor deficits and is estimated to affect 0.5% to 5% of children globally [1–3]. The greatest risk for congenital CMV infection occurs when a mother experiences an infection during pregnancy and the virus disseminates from the site of entry (most likely the oral/nasal cavity) to the fetus [2,3]. However, infection of the fetus in this circumstance is not universal. In fact, only ~40% of primary infections during pregnancy result in congenital infection[4], although the reasons for these drastically different outcomes are unknown. Thus, an understanding of the host/pathogen relationship that governs viral dissemination from the site of entry is critical for the development of effective anti-viral strategies and vaccines.

Primary CMV infection in immune-competent hosts is usually clinically silent, which makes early natural infection difficult to detect and study. Several excellent animal models have been described for investigating CMV infections, including murine (M)CMV, which has been extensively used for *in vivo* studies due to the wealth of available tools. Recent work has identified the nasal mucosa of mice as a potential natural site of primary MCMV infection [5]. The oral/nasal mucosa is also a likely site of entry in humans [6–11], making it an important area for investigation. After entry, CMV must disseminate to sites of shedding including the salivary gland and kidney. For MCMV, the salivary gland is the key site of viral persistence and shedding for transmission to new hosts. Thus, understanding the host/pathogen interactions surrounding MCMV dissemination from the nasal mucosa to the salivary gland should provide key information about natural CMV dissemination after primary infection.

It is generally accepted that myeloid cells are important for dissemination of MCMV [12–18]. In theory, this should expose the virus to cell-mediated immunity and necessitate viral immune evasion for dissemination. However, although there is substantial evidence that myeloid cells *can* carry the virus, it is still unknown whether they are *required*, and the need for immune evasion during this process remains unclear.

Much of the work supporting the conclusion that myeloid cells mediate MCMV dissemination comes from a foot-pad (f.p.) infection model utilizing MCMV that lacks the viral chemokine MCK-2 [12,18]. MCK-2 is a chemokine known to recruit myeloid cells [18–21], and may also play a role in infection of myeloid cells through its presence in the viral fusion complex [22,23]. Infection with an MCK-2 mutant virus results in reduced recruitment of patrolling (CX3CR1^+^) and inflammatory (CCR2^+^) monocytes to the site of infection, a reduced patrolling monocyte-associated viremia, and reduced viral titers in the salivary gland [12,18,21,24]. However, the *kinetics* of initial infection of the salivary gland were identical between wild-type and mutant viruses [21], which would not be expected if dissemination per se was disrupted. Moreover, MCK-2 mutant viruses were more efficiently controlled in the spleen and liver in a NK cell and T cell dependent manner [25] and they provoked stronger T cell responses, in part because fewer CCR2+ inflammatory monocytes were recruited to the site of infection [24]. Thus, MCK-2 mutant viruses reach the salivary gland with similar kinetics at lower titers and appear to be attenuated in ways that are not associated with dissemination.

Evidence for myeloid cell-associated viremia also comes from work with wild-type viruses in mice lacking CX3CR1 [12]. In these mice, wild-type MCMV titers were reduced in the salivary gland after f.p. infection, but not intraperitoneal (i.p.) infection, suggesting that CX3CR1^+^ patrolling monocytes were important for dissemination from the foot pad. This work clearly demonstrated that patrolling monocytes were infected by MCMV and were able to bring MCMV to the salivary gland. However, it was again not clear that they were *required*, as the kinetics of initial infection of the salivary gland were identical in the presence or absence of CX3CR1^+^ patrolling monocytes and only the amplification of MCMV within the salivary gland was impaired in the absence of CX3CR1. The authors speculated that patrolling monocytes may be needed for the virus to reach the salivary epithelium, but not the brown fat surrounding the salivary tissue, implying that there may be alternative means for MCMV to reach the salivary gland brown fat from the foot pad. It is also worth noting that cell-associated viremia was only evident after i.p. infection in these experiments and that CX3CR1-knockout mice had elevated numbers of inflammatory monocytes at the site of infection, which may alter the local immune control and viral spread.

More recent work has utilized the intranasal (i.n.) route of infection. The Stevenson lab has identified the nasal mucosa as a natural portal of entry for vertical transmission of MCMV [5,13], making the i.n. infection model highly relevant to natural MCMV infection. Interestingly, the data suggested that dendritic cells, and not patrolling monocytes, were important for viral dissemination to the salivary gland after infection of the nasal mucosa [13]. This work also showed that viral dissemination was dependent on the hyaluronin binding protein CD44, which implies a cell-associated dissemination, although it is still unclear whether this pathway was required. In fact, these authors also demonstrated the presence of viral DNA in the plasma of infected BALB/c mice after i.p. and i.n. inoculation, implying that infected myeloid cells may not be essential[13], and subsequently confirmed that neither viral MCK-2, nor host CCL2 or CX3CR1 were necessary for viral dissemination[26]. Collectively, these data suggest that myeloid cell-mediated dissemination occurs, however, whether productive infection of these cells is required for dissemination is not known.

If MCMV travels by cell-associated viremia, it should be exposed to cell-mediated immunity. All CMVs encode several genes to modulate T cell and NK cell responses and it remains an open question whether these genes facilitate viral dissemination from the portal of entry [27]. For MCMV there are 3 known genes that modulate MHC-I levels on the surface of infected cells (m04, m06 and m152) [27–29]. The protein products of m06 and m152 reduce MHC-I levels, thereby impairing CD8^+^ T cell recognition of infected cells. The m152 protein product also antagonizes the expression of RAE-1 ligands for NKG2D, which is an important activation molecule for NK cells [30–32]. Finally, the m04 protein product rescues some MHC-I expression to avoid “missing-self” activation by NK cells [33]. Thus, these genes collectively protect infected cells from killing by CD8^+^ T cells and NK cells [30–32,34–36]. After i.p., i.v. and f.p. infections, deletion of all 3 genes in various combinations, consistently impairs acute viral replication in an NK cell- [31,32,37,38], and CD8^+^ T cell-dependent manner [39–41]. However, even in the absence of all 3 evasion genes, the impact on viral fitness *in vivo* was modest–MCMV still spread systemically and replicated, just to a lesser degree. Moreover, Reddehase and colleagues noted several years ago that loss of these 3 evasion genes led to diminished viral transcription in the draining lymph nodes after f.p. infection and suggested that perhaps impaired dissemination might account for some of the reductions in viral titers elsewhere in the body [38]. Thus, although the immune evasion genes m04, m06 and m152 have a profound impact *in vitro*, their impact *in vivo* has been consistent but less dramatic, and it remains unclear how they contribute to viral dissemination.

The goal of this study was to investigate the need for infection of hematopoietic cells after i.n. infection using a novel recombinant strain of MCMV that could not replicate in hematopoietic cells, and to test whether such viral dissemination is regulated by immune responses and dependent on immune evasion. Our data not only confirm that infected hematopoietic cells disseminate MCMV after i.n. infection, but also show for the first time that such dissemination is essential in the presence of strong NK cell responses, but not when NK cells or T cells were depleted. Thus, NK cells and T cells could control dissemination that occurred independently of hematopoietic cell infection, but failed to control hematopoietic cell-mediated dissemination. Furthermore, virus evasion of T cells and NK cells was required to support hematopoietic cell-mediated dissemination and productive infection of the salivary gland. Overall, these data argue that hematopoietic cell-associated dissemination after i.n. infection is a restriction point for MCMV that can be targeted by cell-mediated immune mechanisms, necessitating viral modulation of NK and T cell responses for productive dissemination.

## Results

### MCMV infection of hematopoietic cells is required for dissemination in C57BL/6 mice after i.n. inoculation

**Fig 1:**
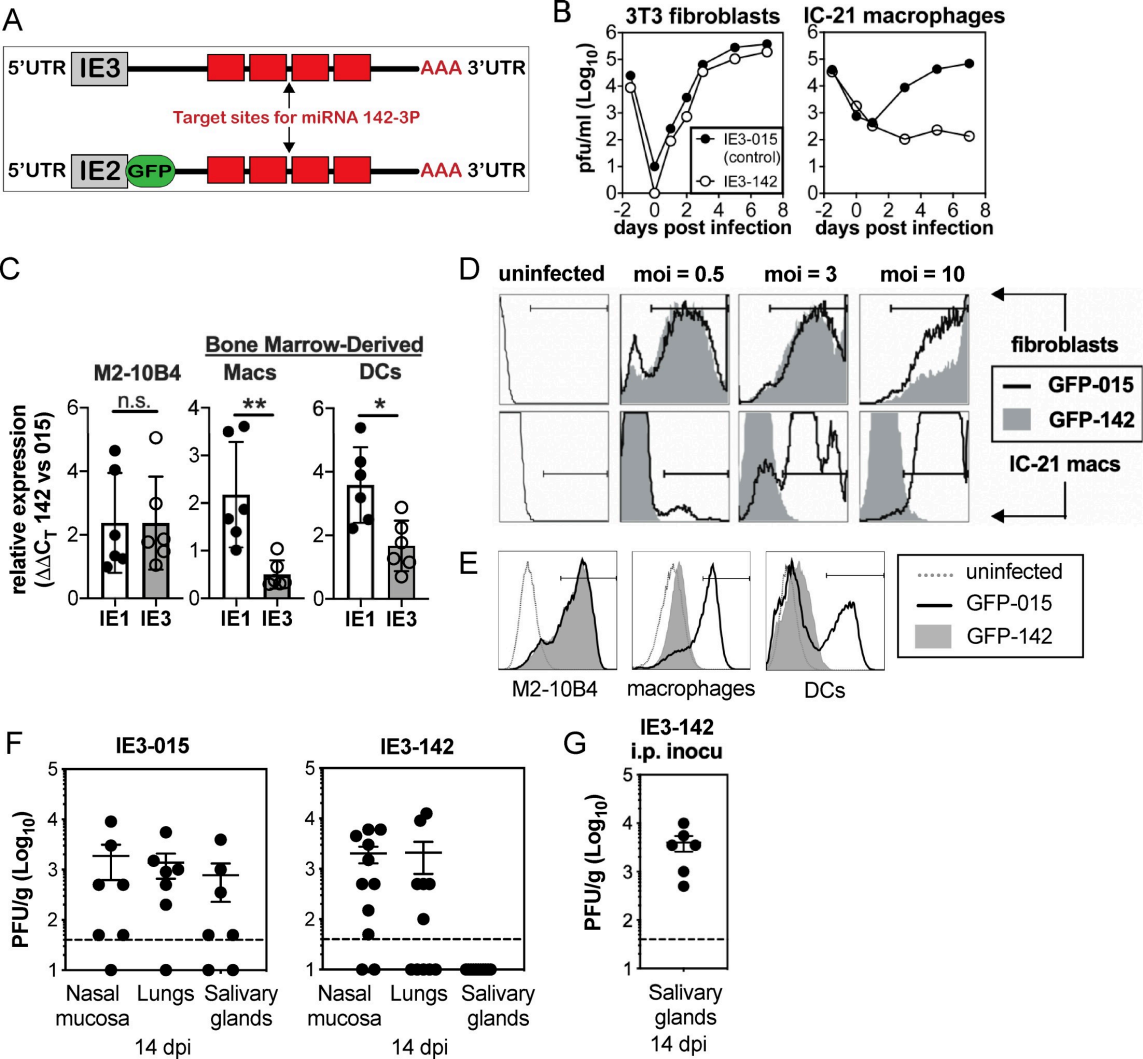
MCMV must use infected hematopoietic cells for dissemination in C57BL/6 mice after i.n. inoculation. **A.** Schematic of miR-142-3p targeted viruses (MCMV-IE3-142 and MCMV-GFP-142). Four target sites for miR-142-3p were inserted into the 3’ untranslated region of the essential viral gene IE3 or into a GFP-SIINFEKL fusion construct inserted into the IE2 locus. Control viruses contain shuttle vector sequences without miR-142-3p target sites in the same location. **B.** Targeting IE3 with miR-142-3p binding sites prevents viral replication in miR-142-3p-expressing macrophages. Multi-step growth curves of the MCMV-IE3-015 control virus and the MCMV-IE3-142 virus in 3T3 fibroblasts and IC-21 macrophages. **C.** The miR-142-3p targets IE3 gene and inhibits its gene expression. IE1 and IE3 gene expression were evaluated by RT-PCR after infection of M2-10B4 cells and bone marrow-derived macrophages and dendritic cells with either MCMV-IE3-015 or MCMV-IE3-142 viruses. Gene expression was normalized to β-actin in each sample and compared between MCMV-IE3-142 and MCMV-IE3-015 infected samples using the ΔΔC_T_ method. The bar graph represents the mean value. Each symbol represents an individual cell sample, and error bars represent the standard deviation. Data are from one representative experiment of three independent experiments. **D-E.** Targeting IE3 with miR-142-3p binding sites inhibits gene expression. Regulation of gene expression by miR-142-3p is visualized by GFP expression after infection of 3T3 fibroblasts and IC-21 macrophages (**D**) or M2-10B4 cells and bone marrow-derived macrophages and dendritic cells (**E**) with either MCMV-GFP-015 or MCMV-GFP-142 viruses. **F-G.** Productive infection of hematopoietic cells is necessary for viral dissemination after i.n. inoculation, but not after i.p. inoculation. Virus titers in the nasal mucosa, lungs and salivary glands at 14 days after i.n. inoculation (**F**) or i.p. inoculation (**G**), with 10^6^ PFU MCMV-IE3-142, or control virus MCMV-IE3-015. Each symbol represents an individual animal. The solid line shows the mean titer, and error bars represent the SEM. Dashed lines show the detection limit (50 PFU/g). Data are combined from two independent experiments.

MCMV has been proposed to disseminate from the nasal mucosa and other tissues in hematopoietic cells [13,42]. To test whether infection of hematopoietic cells is necessary after intranasal infection, we constructed a recombinant MCMV containing four repeated targeting sites for the microRNA miR-142-3p in the 3’ untranslated region of the essential viral gene IE3 (MCMV-IE3-142, Fig 1A). As miR-142-3p is exclusively and uniformly expressed in hematopoietic cells, we predicted that MCMV-IE3-142 would fail to replicate in hematopoietic cells due to targeting of IE3 expression by miR-142, but would replicate to wild type levels in all other cell types. A similar strategy was recently shown to successfully inhibit human (H)CMV gene expression in myeloid cells[43]. As a control, a second virus was produced containing shuttle vector sequences, but no miR-target sites (MCMV-IE3-015). While both viruses replicated equally well in 3T3 fibroblast cells, only the control virus replicated in IC-21 macrophages, while replication of MCMV-IE3-142 was completely blocked, demonstrating the effectiveness of the hematopoietic attenuation (Fig 1B). We also tested the efficacy of this approach in primary bone marrow-derived macrophages and dendritic cells, both of which express equivalent levels of the miR-142-3p (S1A Fig). The miR-142-3p target sequences inhibited IE3 gene expression and resulted in an accumulation of IE1 transcript in cultured macrophages and dendritic cells, but not M2-10B4 fibroblasts (Fig 1C). The accumulation of IE1 transcript is a hallmark of IE3 inhibition for MCMV and IE2 inhibition for HCMV[43–45]. Thus, the miR-142-3p target sites functionally suppressed IE3 gene expression and viral replication in hematopoietic cells.

To directly visualize the regulation of gene expression by miR-142-3p, a second set of viruses was produced containing GFP in the IE2 locus and either 4 miR-142-3p targeting sites (MCMV-GFP-142) or control vector sequences in the 3’ untranslated region (MCMV-GFP-015) (Fig 1A). Both viruses expressed GFP in 3T3 fibroblast cells (Fig 1D) and M2-10B4 fibroblast cells (Fig 1E), but only the control virus expressed GFP in IC-21 macrophages (Fig 1D), or primary bone marrow dendritic cells and macrophages (Fig 1E). Collectively, these *in vitro* data showed that miR-142-3p targeting sites severely limited expression of the targeted viral genes and that targeting the essential IE3 gene by miR-142-3p markedly inhibited viral replication in hematopoietic cells.

Recent work has shown that the nasal mucosa is a natural site of MCMV entry [5]. Thus, we infected C57BL/6 mice by the intranasal route with either MCMV-IE3-142 or control viruses (MCMV-IE3-015). This approach has been previously shown to infect the olfactory neurons (an epithelial cell) in the nasal mucosa, and alveolar macrophages and epithelial cells in the lungs [5,13,42,46,47]. Both viruses replicated in the nasal mucosa and lungs. However, two weeks after infection, the MCMV-IE3-142 virus was undetectable in the salivary gland, unlike the control virus (Fig 1F) and viral genome copies in the salivary gland were markedly reduced (S1B Fig). Similar results were obtained after footpad inoculation, which is considered reflective of infection via licking skin abrasions or biting, another possible natural route of infection for MCMV (S1C Fig). In contrast, infection by the i.p. route, which allows hematogenous spread of cell-free virus [48], enabled MCMV-IE3-142 to replicate robustly in the salivary gland (Fig 1G). Taken together, these data show that infection of hematopoietic cells is essential for efficient viral spread to salivary gland after intranasal infection.

### NK cells and T cells work together to modulate virus dissemination

**Fig 2:**
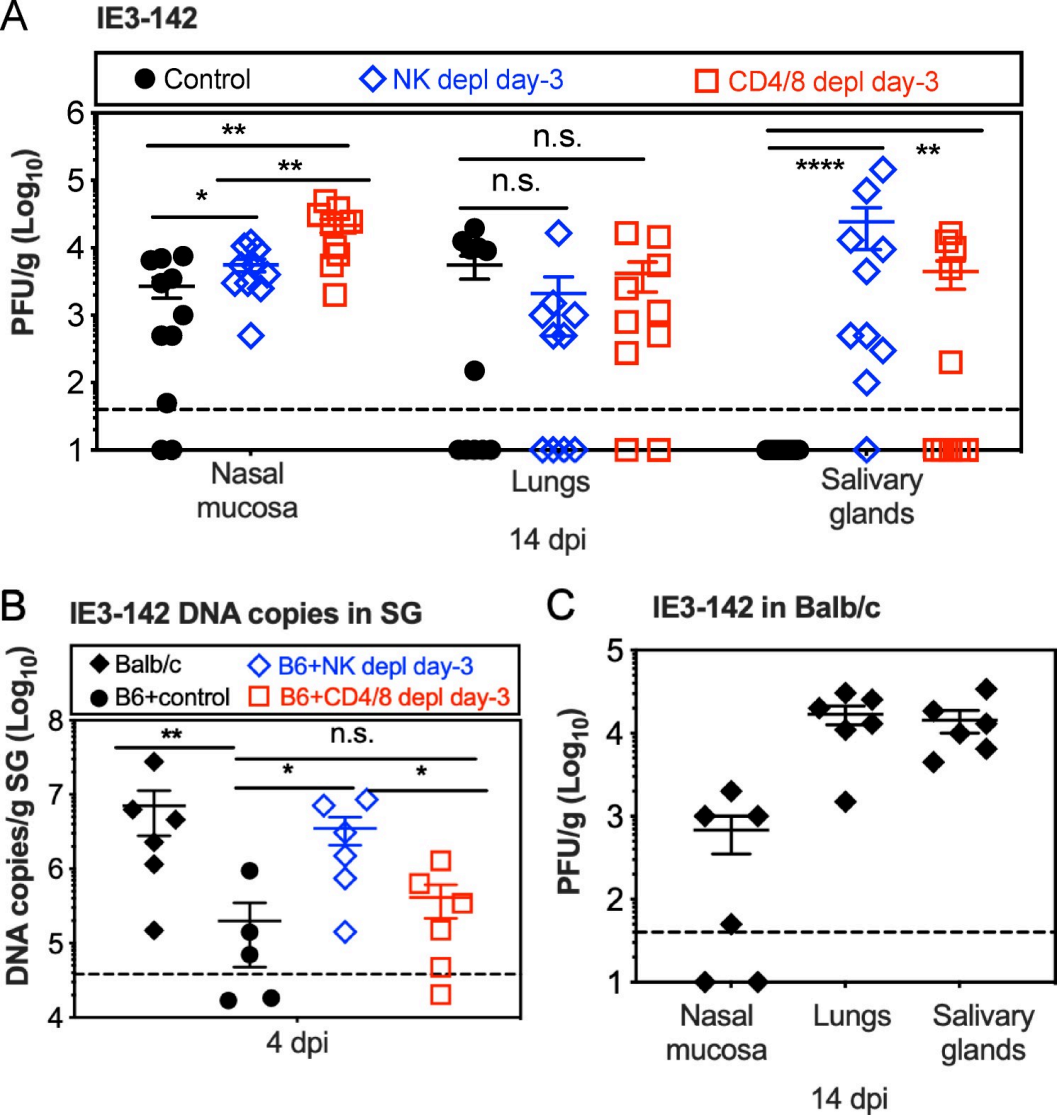
NK cell and T cell work together to modulate virus dissemination. **A.** Depletion of NK cells or T cells from C57BL/6 mice enables MCMV-IE3-142 dissemination from the nasal mucosa to the salivary gland. C57BL/6 mice were depleted of NK cells or CD4^+^ T cells and CD8^+^ T cells before i.n. infection with MCMV-IE3-142. Shown are the viral titers in the nasal mucosa, lungs and salivary gland 14 days after infection. Each symbol represents an individual animal. The solid line shows the mean value, and error bars represent the SEM. Dashed lines show the detection limit (50 PFU/g). Data are combined from at least two independent experiments. **B.** Early viral dissemination is affected by NK cell responses. C57BL/6 mice with or without depletion of either NK cells or CD4^+^ and CD8^+^ T cells were i.n. inoculated with MCMV-IE3-142. Shown are viral DNA copies in the salivary gland at 4 dpi. Data are displayed as in **A** and are combined from two independent experiments. **C.** MCMV does not require infection of hematopoietic cells to reach the salivary glands after i.n. infection of BALB/c mice. Shown are virus titers in the nasal mucosa, lungs and salivary glands of BALB/c mice at 14 days after i.n. inoculation of MCMV-IE3-142. Data are displayed as in **A** and are combined from two independent experiments.

Our data indicate that MCMV dissemination after i.n. inoculation required the productive infection of hematopoietic cells in C57BL/6 mice. Given this need to use infected cells for dissemination, we speculated that cell-mediated immune responses might modulate this process. Thus, we next tested whether NK cells or T cells regulated dissemination. To this end, NK cells or T cells were depleted prior to i.n. infection with MCMV-IE3-142. Depletion of each cell type was confirmed by flow cytometry of cells in the blood prior to infection as well as each week during the experiment (S2 Fig). Remarkably, depletion of NK cells allowed MCMV-IE3-142 to reach the salivary gland in nearly all (9 of 10) mice, while only slightly increasing viral titers in the nasal mucosa itself. In contrast, depletion of CD4^+^ and CD8^+^ T cells increased viral titers in the nasal mucosa more markedly, but had a more modest effect on MCMV-IE3-142 reaching the salivary gland, with 5 of 10 mice showing detectable viral titers by day 14 (Fig 2A). There was no correlation between viral titers in the nasal mucosa and the salivary gland (S3 Fig), suggesting that increased replication at the sites of entry did not account for the ability of MCMV-IE3-142 to reach the salivary gland. Depletion of NK cells increased the quantities of viral genomes in the salivary gland as early as day 4 after infection, which was not the case in T cell depleted mice (Fig 2B). As this time point precedes infection of salivary epithelial cells after i.n. inoculation [13], these data imply that NK cells regulated the efficiency and/or route of acute dissemination to the salivary gland. These experiments were performed with viruses built on the original MCMV BAC strain (MW97.01 [49]), which is known to have a point mutation in MCK-2 that attenuates salivary gland growth [50]. Importantly however, an independently-generated virus expressing a repaired MCK-2 and harboring miR-142-3p target sites in the IE3 locus (S4A Fig), was still impaired in reaching the salivary gland after i.n. infection, unless NK cells were depleted (S4B and S4C Fig). Furthermore, we infected BALB/c mice with the original MCMV-IE3-142, harboring the mutation in MCK-2. BALB/c mice are susceptible to MCMV due to a lack of Ly49H^+^ NK cells (among other differences) and thus do not control acute MCMV replication as efficiently as C57BL/6 mice. In this setting, without strong NK cell responses, genome copies of MCMV-IE3-142 in the salivary gland on day 4 were comparable to NK-depleted C57BL/6 mice (Fig 2B) and viral replication was evident by day 14 in all mice (Fig 2C). Thus, the MCK-2 mutation cannot account for the reduced salivary gland infection by MCMV-IE3-142. Rather, these data suggest that early MCMV dissemination from the nasal mucosa can occur by hematopoietic cell-dependent or -independent means. Strong NK cell responses, and to a lesser extent T cell responses, controlled the non- hematopoietic cell dissemination and thus forced MCMV to use infected hematopoietic cells, which was selectively inhibited by the miR-142-3p target sites.

### Early dissemination of MCMV requires evasion of cell-mediated immunity and is restored by NK cell depletion

**Fig 3:**
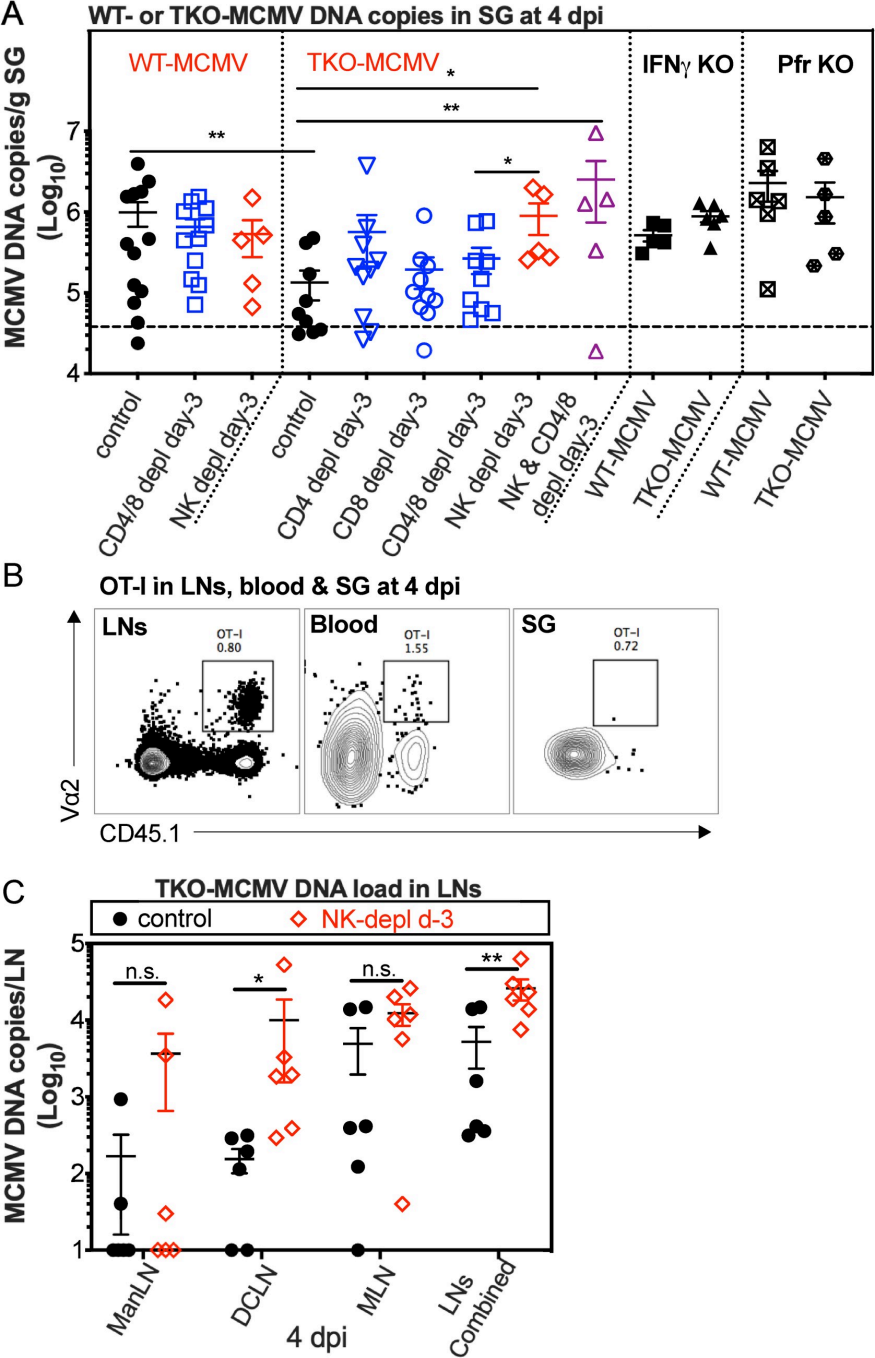
Early dissemination of TKO-MCMV is restored by NK cell depletion. **A.** NK cells prevent early dissemination of TKO-MCMV to the salivary gland after i.n. infection of C57BL/6 mice in a manner dependent on both IFN-γ and perforin. C57BL/6 mice depleted of the indicated cells before i.n. infection or mice lacking IFN-γ or perforin were intranasally inoculated with either WT-MCMV or TKO-MCMV. Shown are viral DNA copies in the salivary gland 4 days after infection. Each symbol represents an individual animal. The solid line shows the mean value, and error bars represent the SEM. Dashed lines show the detection limit. Data are combined from at least two independent experiments for each condition. **B.** MCMV-specific T cells are not present in the salivary gland by day 4 after i.n. infection. Representative FACS plots show OT-I cells in the blood, draining LNs (ManLNs, DCLNs and MLNs) and salivary gland 4 days after i.n. infection with MCMV-Ova. Data show cells in one representative mouse from one experiment. **C.** NK cell depletion increases the TKO-MCMV DNA loads in draining LNs (ManLNs, DCLNs and MLNs). NK cells were depleted or not from C57BL/6 mice beginning 3 days prior to infection. Shown are viral DNA copies in the indicated lymph nodes individually or combined at 4 days post infection. Data are displayed as in **A** and are combined from 2 independent experiments.

Our data suggest that the virus would need to evade NK cell and/or T cell recognition during infection of hemopoietic cells for efficient dissemination to the salivary gland after i.n. infection. Since infection of hematopoietic cells was required for dissemination in the presence of NK cells and T cells in C57BL/6 mice (Fig 2), we addressed whether viral evasion of either cell type was critical for dissemination after i.n. infection. MCMV encodes three genes (m04, m06 and m152) that modulate responses by both NK cells and T cells via altering MHC-I levels and NKG2D ligands [27–32]. If these evasion genes are required during dissemination, we reasoned that we would detect reduced quantities of TKO-MCMV DNA in the salivary gland, which should be rescued by depletion of T cells and/or NK cells. To test this, C57BL/6 mice were infected i.n. with either wild-type MCMV (WT-MCMV) or “Triple Knockout” MCMV (TKO-MCMV) lacking all three evasion genes, and assessed viral DNA copies in the salivary gland on day 4 after infection. Indeed, the TKO-MCMV DNA load was approximately 10-fold reduced in the salivary gland on day 4 after infection compared to WT-MCMV in unmanipulated C57BL/6 mice (Fig 3A). However, when CD8^+^ T cells, CD4^+^ T cells or both CD4^+^ and CD8^+^ T cells were depleted, TKO DNA load was only marginally increased (about 2-fold) and this did not reach significance (Fig 3A). At this day 4 time point, virus-specific CD8^+^ T cells can be detected in draining lymph nodes (mandibular LNs, deep cervical LNs and mediastinal LNs [51,52]), but not yet in the salivary gland (Fig 3B). Thus, these data suggest that T cells are not principally responsible for the defect in dissemination of TKO-MCMV. However, depletion of NK cells alone or NK cells and T cells from C57BL/6 mice resulted in complete restoration of early TKO-MCMV dissemination to the salivary gland (Fig 3A). Moreover, elevated TKO-MCMV DNA was evident at this time in the draining lymph nodes in the absence of NK cells (Fig 3C). These data are consistent with the previous results from the Reddehase lab[38] suggesting that m04, m06 and/or m152 may influence viral dissemination. Importantly, mice deficient in either perforin or IFN-γ completely failed to restrict TKO-MCMV dissemination (Fig 3A). Thus, our data suggest that NK cells strongly limited the dissemination of TKO-MCMV through a mechanism that required both perforin and IFN-γ. Together, these data show that the m04, m06 and m152 evasion genes are required to escape NK cell responses during acute dissemination in hematopoietic cells.

### Evasion of MHC-I antigen-presentation and CD8^+^ T cells is critical for viral persistence at sites of entry and replication in the salivary gland

**Fig 4:**
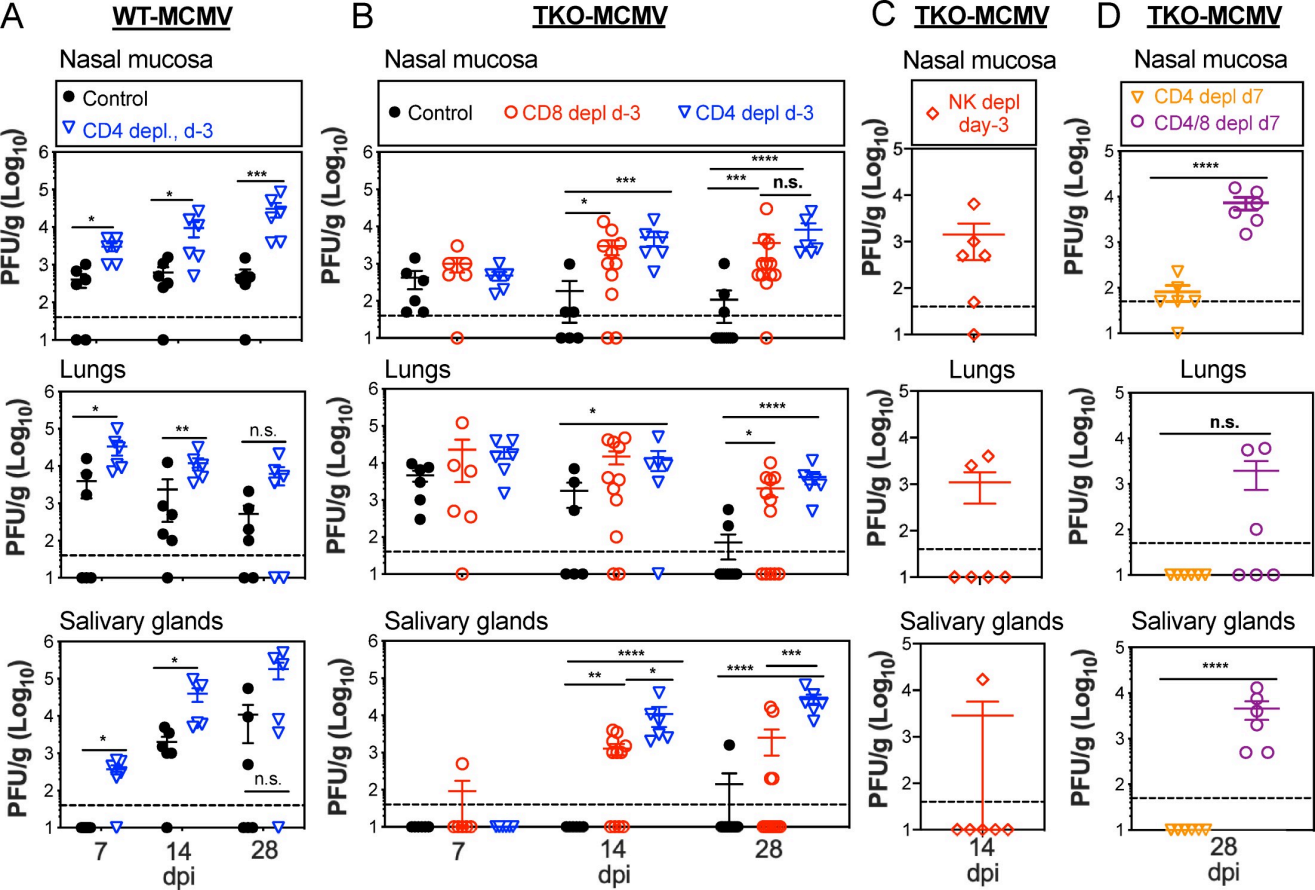
Evasion of MHC-I antigen-presentation and CD8^+^ T cells is critical for viral persistence at site of entry and replication in the salivary gland. **A.** WT-MCMV persists in the nasal mucosa and spreads to the salivary gland within 14 days after i.n. infection. Virus titers in the nasal mucosa, lungs and salivary glands of C57BL/6 mice with or without CD4^+^ T cells depletion before infection at 7, 14 and 28 days after i.n. inoculation of WT-MCMV. Each symbol represents an individual animal. The solid line shows the mean titer, and error bars represent the SEM. Dashed lines show the detection limit (50 PFU/g). Data are combined from two independent experiments. **B.** Lack of MHC-I evasion genes prevents viral persistence in the nasal mucosa and spread to the salivary gland. Viral titers in the nasal mucosa, lungs and salivary gland 7, 14 and 28 days after i.n. inoculation of C57BL/6 mice infected with TKO-MCMV. Infected mice were treated with an isotype control antibody or depleted of CD4^+^ or CD8^+^ T cells before infection. Data are displayed as in **A** and are combined from at least two independent experiments. **C.** NK cell depletion before infection does not rescue TKO-MCMV replication in the salivary glands after i.n. infection of C57BL/6 mice. Shown are virus titers in the nasal mucosa, lungs and salivary glands at 14 days post infection. Data are displayed as in **A** and are combined from two independent experiments. **D.** CD8^+^ T cells can control TKO-MCMV if they are primed in the presence of CD4^+^ T cell help. Virus titers in the indicated organs at day 28 post infection are shown. C57BL/6 mice were depleted of either CD4^+^ T cells or both CD4^+^ and CD8^+^ T cells, beginning at day 7 after i.n. infection of TKO-MCMV. Data are displayed as in **A** and are combined from two independent experiments.

Our data suggest that viral evasion of NK cells is critical for efficient hematopoietic cell-mediated dissemination of MCMV to the salivary gland from a mucosal site of infection, as measured by the appearance of viral DNA. However, the data suggest that TKO-MCMV DNA still reached the salivary gland at reduced levels, after which we would expect viral infection of the acinar and ductal epithelial cells and production of infectious particles. Therefore, we next tested whether viral replication in the salivary gland was also affected by NK cells, T cells or both. While WT-MCMV replicated at the entry sites (nasal mucosa and lungs) from 7 days after infection until the end of the experiment at day 28, and also spread to salivary gland within 14 days (Fig 4A), TKO-MCMV was rapidly controlled at the sites of entry and failed to replicate in the salivary gland except in one mouse (Fig 4B). Importantly, depletion of CD8^+^ T cells prior to infection (Fig 4B), but not NK cells (Fig 4C), enabled TKO-MCMV to replicate normally and persist for at least 28 days in the nasal mucosa and lungs while also becoming detectible in salivary gland with similar kinetics as WT-MCMV. These data clearly show that, while evasion of NK cells enabled efficient dissemination of MCMV to the salivary gland (Fig 3), evasion of MHC-I antigen presentation and CD8^+^ T cells, was crucial for MCMV to persist in the nasal mucosa and lungs and to effectively replicate in the salivary gland after arrival (Fig 4).

Surprisingly, depletion of CD4^+^ T cells enabled increased viral replication in the nasal mucosa, lungs and salivary gland for both WT-MCMV and TKO-MCMV, completely reversing the restriction on replication of TKO-MCMV in each site and enabling persistently elevated titers of both viruses (Fig 4A and 4B). CD4^+^ T cells are well-known to play a direct role in the control of MCMV in the salivary gland [53–55] and a recent report has suggested that MCMV must evade CD4^+^ T cells via the viral gene M78 for efficient replication in the salivary gland [56]. However, since none of the three viral genes missing in TKO-MCMV (m04, m06 and m152) are known to contribute to evasion of MHC-II or CD4^+^ T cells, it seemed unlikely that the defect in TKO-MCMV spread (Fig 3A) could be due to a failure to evade anti-viral CD4^+^ T cells. Therefore, we considered whether CD4^+^ T cell help was needed to develop functional CD8^+^ T cell responses after i.n. infection, rather than to directly control TKO-MCMV. This would be unexpected since previous work suggested that CD4^+^ T cell help plays only a modest role in supporting CD8^+^ T cells after i.p. infection, primarily affecting T cell recall capacity and memory inflation [57,58]. To test this, we depleted CD4^+^ T cells beginning at day 7 after infection, which should allow CD8^+^ T cells to be primed in the presence of CD4^+^ T cell help. At this time-point, similar titers of WT-MCMV and TKO-MCMV were present in the nasal mucosa and lungs, but neither virus was replicating in the salivary gland (Fig 4B). This delayed depletion of CD4^+^ T cells restored control of TKO-MCMV in the nasal mucosa and lungs and prevented any virus from being detected in the salivary gland (Fig 4D). Control was not due to the effects of CD4^+^ T cells in the first week of infection because double depletion of CD4^+^ T cells and CD8^+^ T cells, both beginning on day 7 after infection, restored TKO-MCMV replication in all tissues (Fig 4D). Thus, delayed depletion of CD4^+^ T cells restored control of TKO-MCMV in a CD8^+^ T cell-dependent manner.

**Fig 5:**
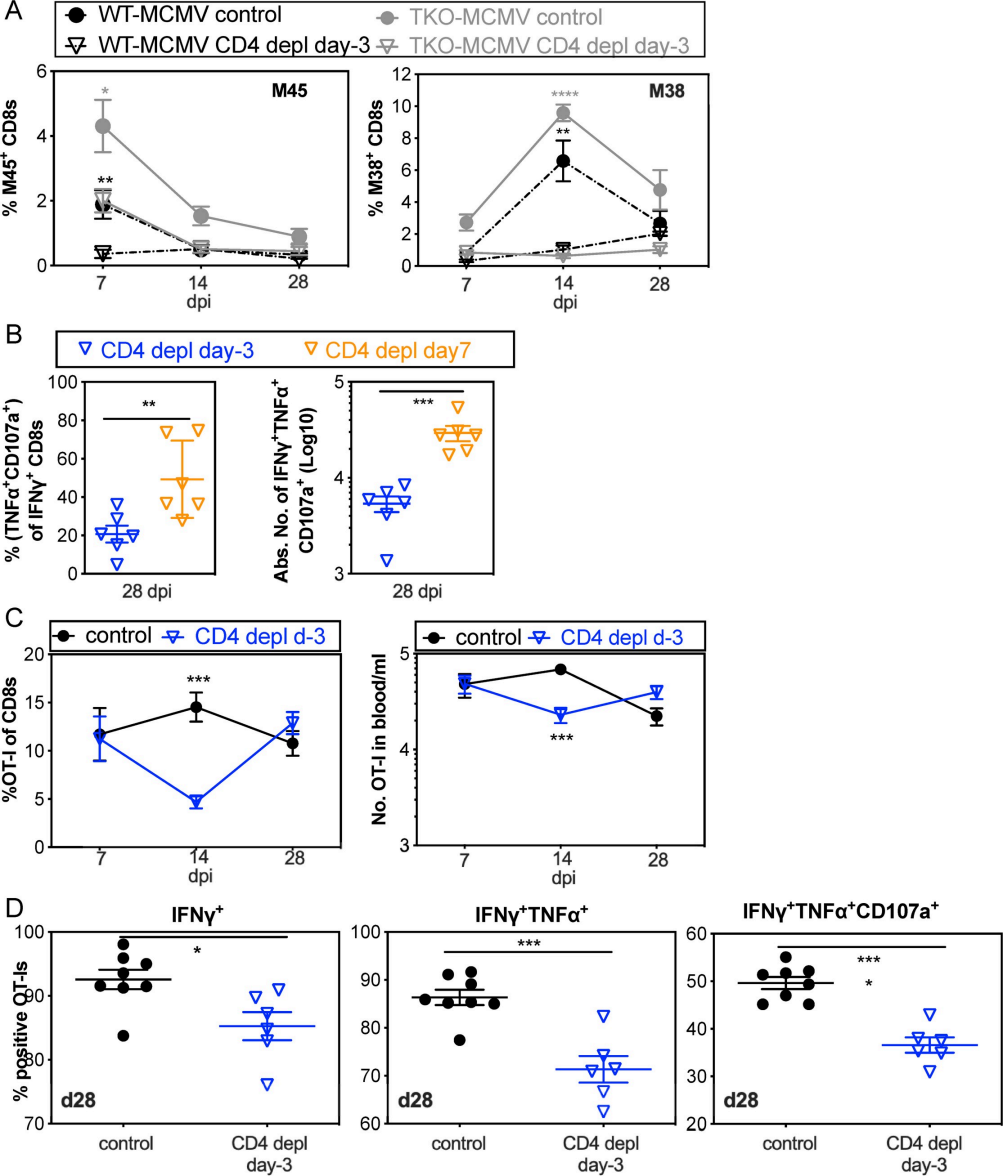
CD4^+^ T cell help is needed for the proliferation and function of MCMV-specific CD8^+^ T cells. **A.** CD8^+^ T cells are reduced in frequency after i.n. infection in the absence of CD4^+^ T cell help. Shown is the frequency of viral tetramer-specific CD8^+^ T cells in the blood of recipients at the indicated time points with or without CD4^+^ T cell depletion before infection. Data show the average frequency of T cells at day 7 (n = 9–12), day 14 (n = 6–9) and day 28 (n = 3–6) after i.n. infection and are derived from one representative experiment of at least 3 independent experiments. **B.** CD8^+^ T cell function is impaired in the absence of CD4^+^ T cell help after i.n. infection, but improved by delaying CD4^+^ T cell depletion until day 7. Each symbol represents an individual animal. The solid line shows the mean value, and error bars represent the SEM. Data are combined from two independent experiments. **C.** The frequency and number of OT-Is are impaired in the absence of CD4^+^ T cell help after i.n. infection with MCMV-Ova. CD4^+^ T cells were depleted or not from C57BL/6 mice. One day before i.n. infection with MCMV-Ova, mice received 5000 OT-I T cells. Shown are the frequency (left) and absolute number (right) of OT-I cells in blood over time after infection. Data show the average values from 6–8 animals per group, and error bars represent the SEM. CountBright absolute counting beads (ThermoFisher Scientific) were included to determine the number of cells in the blood. **D.** The function of OT-I T cells in the spleen is impaired on a per-cell basis in the absence of CD4^+^ T cell help. OT-I T cells in the spleens of adoptive recipients (as described in **C.**) were analysed for production of IFN-γ, TNF-α and degranulation (exposure of CD107a) after stimulation with SIINFEKL peptide 28 days after infection. Each symbol represents an individual animal. The solid line shows the mean value, and error bars represent the SEM.

Consistent with a role for CD4^+^ T cell help for CD8^+^ T cells, CD4^+^ T cell depletion prior to i.n. infection significantly reduced the frequency and number of MCMV-specific CD8^+^ T cells in the blood, sharply impairing the normally dominant responses against the M45 and M38 antigens (Fig 5A). In contrast, delaying depletion of CD4^+^ T cells until day 7 after infection significantly increased the frequency and number of functional CD8^+^ T cells (Fig 5B). For precise quantitation of CD8^+^ T cell function per cell we used OT-I T cells stimulated by i.n. infection with MCMV-Ova. Depletion of CD4^+^ T cells prior to infection resulted in a transient reduction in OT-I frequency and number (Fig 5C) and a sustained impairment of OT-I cytokine production and degranulation (Fig 5D). Thus, CD4^+^ T cell help was critical for the development of functional CD8^+^ T cells after i.n. inoculation, which inhibited the replication of TKO-MCMV.

In sum, our data suggest the following model: 1) MCMV can disseminate by both hematopoietic cell-dependent and -independent means after intranasal infection. 2) NK cells are able to potently limit the non-hematopoietic cell pathway(s) of dissemination and this is evident in C57BL/6 mice where virus lacking the ability to productively infect hematopoietic cells disseminated very poorly. 3) MCMV disseminating within infected hematopoietic cells resisted cell-mediated immunity via the m04, m06 and m152 immune evasion genes, which 4) protected MCMV from NK cells during dissemination and 5) from CD8^+^ T cells in the nasal mucosa and salivary gland. Finally, and unexpectedly, 6) CD4^+^ T cells were required for the development of functional CD8^+^ T cell responses after intranasal infection.

## Discussion

Recent work showed that natural MCMV transmission from infected mothers to pups occurs through the nasal mucosa [5]. Moreover, human CMV is thought to infect via an oral/nasal route [6–11] and it has been proposed that the nasal mucosa may be the dominant site of entry [5]. Very little is known about the immune response or viral dissemination after a nasal infection. We used the i.n. model of infection with a low inoculum volume (20 μl total), which should largely restrict the virus to the nasal mucosa and upper airways [59,60], enabling us to assess viral dissemination from mucosal tissues without a mechanical breach of the barrier. Our data reveal a surprisingly complex and previously unappreciated host/pathogen relationship after intranasal inoculation of mice with MCMV.

First, we tested whether infection of hematopoietic cells was required for dissemination in C57BL/6 mice. There is strong evidence that infected hematopoietic cells bring MCMV to the salivary gland. However, it was unclear whether this route of dissemination is required for MCMV, or one of multiple potential routes of dissemination, especially since MCMV reached the salivary gland with reduced titers but identical kinetics when the viral chemokine MCK-2 was deleted, or in mice lacking CX3CR1^+^ patrolling monocytes, CCR2^+^ inflammatory monocytes, or in which CCL2 was blocked [12,18,21,24,26]. Our data confirm that expression of IE3 in hematopoietic cells was necessary for virus to infect the salivary gland and replicate in C57BL/6 mice. The miR-142-3p target sites in the IE3 gene reduced the numbers of viral genomes detected in the salivary gland within a few days of infection, and replicating virus was not detected (Figs 1 and 2). Remarkably, however, this was not the case if NK cells were depleted or in BALB/c mice (Fig 2). Therefore, our data suggest that the virus is also disseminating via an alternative route, that is not dependent on hematopoietic cell infection, and is only evident in the absence of strong NK cell control. Importantly, our conclusion does not rely on the ability of miR-142-3p to absolutely suppress IE3 expression. Even if there was some leaky expression of IE3 that enabled the MCMV-IE3-142 to replicate in some hematopoietic cells, the overall effect of the miR-142-3p target sites was the profound suppression of viral replication in the salivary gland, strongly arguing that infection of hematopoietic cells is a required step in the presence of strong NK cell responses, but not when NK cells are missing or ineffective. Nevertheless, a potential caveat to this interpretation is that the efficacy of the miR-142-3p target sites might differ in BALB/c mice or following NK or T cell depletion, due to increased virus levels at the site of infection. However, minimal replication was observed in the salivary gland of intact C57BL/6 mice, despite viral titers at the site of inoculation and lungs reaching levels as high or higher than those observed in NK or T cell depleted mice. Moreover, depletion of T cells was associated with less infection of the salivary gland but significantly higher viral titers in the nasal mucosa compared to depletion of NK cells (Figs 2 and S3). Furthermore, our data indicate that suppression of IE3-expression was efficient *in vitro* (where no immune response is regulating the impact of miR-142-3p) and *in vivo* (where MCMV-IE3-142 failed to replicate in the salivary gland). These data suggest that the ability to disseminate is not linked to the acute viral titers in the mucosa. Thus, we hypothesize that MCMV can disseminate by an alternate route, which is well controlled by robust NK cell responses. Our primary hypothesis is that virus travels by cell-free viremia in the absence of NK cell-mediated suppression. In support of this idea, the Stevenson lab has identified viral DNA in the plasma of BALB/c mice after i.p. infection and in very small amounts after i.n. infection [13], which fits with our finding that viral infection of hematopoietic cells is not required in the absence of NK cells in C57BL/6 mice, or in BALB/c mice (Fig 2). As yet, we have been unable to detect cell-free virus in the plasma of NK-depleted C57BL/6 mice at day 4 after infection. An alternative possibility is that infected endothelial cells may be sloughed into the blood stream as they become cytomegalic, a mechanism that was first proposed to mediate dissemination of CMV by Goodpasture and Talbot almost 100 years ago [61]. Thus, future work will be aimed at defining the source of virus that arrives in the salivary gland in each of these settings.

The conclusion that MCMV can use multiple routes for dissemination potentially informs the interpretation of previous work showing that loss of MCK-2 from the virus or CX3CR1 or CCR2 from the mice may reduce, but does not prevent dissemination [12,18,21,24,26]. Indeed, in all 3 cases, loss or reduction of dissemination by hematopoietic cell infection may have been offset by an alternative, non-hematopoietic cell-dependent, pathway of dissemination. The chemokine function of MCK-2 has been suggested to combine with chemokines recruiting CX3CR1^+^ patrolling monocytes to mediate dissemination from the footpad [24]. Moreover, MCK-2 has been proposed to be part of the MCMV fusion complex needed for entry into myeloid cells [22,23]. However, impairing these pathways would not be expected to inhibit dissemination by cell-free viremia or infected endothelial cells sloughed into the blood stream. Thus, future work will need to evaluate the impact of these and other interventions in settings where the route of dissemination is restricted to hematopoietic cells in comparison with settings in which infection of hematopoietic cells is blocked (i.e. using the miR-142-3p targeting system).

The difference between C57BL/6 and BALB/c mice is very interesting. It is well known that different mouse strains are resistant to MCMV in a manner that is dependent on NK cells, including C57BL/6, Ma/My, NZW, BALB.K and PWK/Pas [62–68] and humans may display a similar range of resistant or sensitive NK cell phenotypes in response to HCMV infection. C57BL/6 mice are paradigmatic of a “resistant” genetic background due to expression of the activating receptor Ly49H, which recognizes viral m157 [69,70]. However, there are multiple strain differences between these animals and a larger study will be needed to determine if this effect is exclusively due to Ly49H and whether other resistant strains also restrict MCMV to hematopoietic cell-mediated dissemination.

In the current study, we reasoned that evasion of T cells and NK cells would become paramount if MCMV could only disseminate within infected cells. Indeed, we found that MCMV lacking m04, m06 and m152 reached the salivary gland poorly by day 4 after i.n. infection where it was unable to replicate in C57/BL6 mice. While previous work demonstrated attenuation of MCMV lacking these 3 evasion genes, our data are the first to describe a vital role for m04, m06 and m152 during viral dissemination and the first to show a catastrophic loss of viral fitness in the absence of these genes. In contrast, previous work described more subtle reductions in viral fitness as a result of deleting these genes, including reduced acute viral titers and latent viral loads, as well as clear improvements in CD8^+^ T cell killing of infected cells in vitro and in vivo [34,38–40,71–73]. Only the replication of MCMV in the salivary glands of CD4^+^ T cell deficient mice and its reactivation from latency in explant cultures have been reported to truly require these evasion genes [41,74]. However, these studies all utilized routes of infection (i.p., i.v. or f.p.) that involve breaking or avoiding a barrier tissue and allow MCMV to directly infect cells in the spleen [48,75,76]. This indicates that at least some MCMV likely had direct access to the blood from the site of inoculation, which may have allowed dissemination without facing cell-mediated immunity. Thus, our data show that cell-mediated immunity can markedly impair viral fitness after infection of mucosal tissues such that the virus was unable to reach the primary site of viral shedding.

Because of the strict requirement for the m04, m06 and m152 evasion genes in C57BL/6 mice, we devoted effort to defining the immune mechanisms that restrict dissemination of MCMV lacking these 3 genes. Acute dissemination from the nasal mucosa to the salivary gland required these evasion genes to limit NK cell control of the virus. Indeed, depletion of NK cells, but not T cells, increased the quantity of viral genomes that were detectable in the salivary gland as early as day 4 post infection (Fig 3). These data are consistent with the hypothesis that NK cells could restrict viral dissemination in infected hematopoietic cells and that evasion of NK cells is vital to protect MCMV during this primary dissemination. Previous work has shown that the m04 and m152 genes contribute to evasion of NK cell responses [30–33,77–79]. The m04 protein complexes with MHC-I and allows increased surface expression of total MHC-I even in cells expressing m152 and m06, to avoid NK cell activation [33]. The m152 gene down-regulates the RAE-1 ligands for the NKG2D activating receptor, consequently inhibiting NK cell responses [31,32,77–79]. Future work will be needed to define the individual roles of viral m04 and/or m152 in improving acute viral dissemination and to extend these studies to include other genes that modulate NK cell function. Together, these data suggest that MCMV’s MHC-I/NKG2D evasion genes play a vital role in avoiding NK cells during hematopoietic cell-mediated spread from mucosal tissues to the salivary gland.

Importantly however, depletion of NK cells did not rescue the *replication* of TKO-MCMV in the salivary gland or nasal mucosa (Fig 4). In contrast, depletion of CD8^+^ T cells restored replication of TKO-MCMV in both tissues. Thus, the m04, m06 and m152 evasion genes protect MCMV from NK cells during dissemination and from T cells within mucosal tissues. These findings are consistent with recent work from the Forster lab indicating that cells infected by MCMV lacking immune-evasion genes are more effectively killed *in vivo* by cytotoxic T cells [73] and with prior work from the Oxenius lab indicating that these 3 immune evasion genes protected MCMV from CD8^+^ T cells in the salivary gland [41].

Our data also unexpectedly revealed a critical role for CD4^+^ T cells after i.n. inoculation. CD4^+^ T cells were previously thought to be essential for controlling MCMV only in the salivary gland, where direct anti-viral functions of the CD4^+^ T cells seem to be required [41,53]. We now show that CD4^+^ T cells were vital for controlling wild-type MCMV in the nasal mucosa and lungs as well, where titers were elevated after intranasal infection (Fig 4A). This was due, at least in part, to an unexpected need for CD4^+^ T cell help for CD8^+^ T cells after i.n. infection. Although CD4^+^ T cell help for CD8^+^ T cells is well established in multiple infectious models (e.g. [80–92]), previous work in the MCMV model had shown that CD4^+^ T cell-deficient mice mounted a remarkably intact CD8^+^ T cell response to MCMV after i.p. or i.v. inoculation [57,58]. However, after i.n. infection, mice lacking CD4^+^ T cells produced few functional anti-viral CD8^+^ T cells and were unable to even limit the dissemination of TKO-MCMV (Figs 4 and 5). Previous work has suggested that MCMV-specific CD8^+^ T cells are primed by cross-presentation after i.p. inoculation [93,94], a route of infection that produces high viral titers in the first few days of infection. It is possible that CD8^+^ T cell priming after i.n. infection relies more heavily on direct presentation. Alternatively, cross-presenting dendritic cells in the draining lymph nodes after i.n. MCMV infection may depend on CD4^+^ T cell help for activation and licensing. Future work will be needed to dissect the specific requirements for CD4^+^ T cells after i.n. infection. In this context, it is interesting to note that the viral M78 gene, which is responsible for reducing MHC-II expression on infected cells, was required for efficient infection of the salivary gland after i.n. inoculation [56].

In summary, our data show that MCMV dissemination can likely occur by multiple routes that are either dependent on, or independent of, hematopoietic cell infection. In the presence of robust NK cell responses MCMV depends on hematopoietic cells to reach the salivary gland, which exposes the virus to cell-mediated immunity, necessitating evasion of NK cell responses for efficient dissemination. Since the intensity of NK cell responses are controlled by host and viral genetics [95], it is intriguing to consider the possibility that different host/pathogen combinations will lead to different routes used by CMV to reach distant tissues. We further confirmed that evasion of CD8^+^ T cells was required for viral replication in the salivary gland, and showed for the first time that this is true in the nasal mucosa as well. These data provide the first *in vivo* evidence for a catastrophic loss of viral fitness in the absence of these immune evasion genes in wild-type, previously uninfected animals. Finally, we found that CD4^+^ T cell help was needed to generate functional CD8^+^ T cells after i.n. infection. Overall, these data suggest that the nasal route of infection places previously unappreciated restrictions on both the virus and the immune response.

## Experimental methods

### Ethics statement

All animals were maintained in the specific-pathogen-free facility of the Thomas Jefferson University and treated in accordance with the AAALAC International regulations (Facility Registration Number: 000443). All animal experiments and procedures were reviewed and approved in protocols 01208 and 01209 by the Thomas Jefferson University Institutional Animal Care and Use Committee, which follows the Office of Laboratory Animal Welfare Public Health Service Policy on Humane Care and Use of Laboratory Animals (Animal Welfare Assurance Number: D16-00051).

### Mice

Six to seven-week old mice were used for all experiments. C57BL/6J mice and BALB/c mice were purchased from the Jackson Laboratory and used directly. OT-I transgenic mice (C57BL/6-Tg(TcraTcrb)1100Mjb/J), CD45.1 mice (B6.SJL-Ptprc^a^ Pepc^b^/BoyJ), IFN-γ knock-out mice (B6.129S7-Ifng^tm1Ts^/J) and perforin knock-out mice (C57BL/6-Prf1^tm1Sdz^/J) were purchased from the Jackson Laboratory and maintained in our animal colony.

### Viruses

Murine cytomegalovirus strains including the wild-type BAC-derived MCMV strain (MW97.01, called WT-MCMV throughout)[49], TKO-MCMV strain (triple knock out of m04, m06 and m152) [72] and MCMV-Ova (which expresses the cognate SIINFEKL peptide) have been previously described [96,97]. They were propagated in M2-10B4 cells as previously described [98].

### Cell culture

M2-10B4 cell line was purchased from ATCC. M2-10B4 cells were cultured in growth media (RPMI-1640 medium with L-glutamine (Mediatech/Cellgro, reference #: 10-040-CV), supplemented with 10% FBS and 100 units/ml Penicillin, 100 μg/ml Streptomycin) at 37°C with 5% carbon dioxide. The bone marrow from femurs and tibias of C57BL/6 mice was used to prepare bone marrow macrophages and dendritic cells. The bone marrow derived macrophages were prepared and cultured as previously described [99]. Dendritic cells were cultured in growth medium including RPMI-1640 medium with L-glutamine supplemented with 10% FBS, 100 units/ml Penicillin, 100 μg/ml Streptomycin, 5ml MEM non-essential Amino Acid solution, 5ml HEPES and 10 ng/ml recombinant mouse GM-CSF (BD Biosciences) as previously described [100].

### Experimental infection

Mice under anesthesia were either infected by the i.n. route with 10^6^ PFU MCMV in 20 μL PBS (10 μL per nare), or by the i.p. route with 10^6^ PFU MCMV in 100 μL PBS. All experiments were approved by the Thomas Jefferson University Institutional Animal Care and Use Committee.

### Generation of miR-142 cell tropism specific MCMV virus

miR-142 virus and control virus were constructed using the λ-derived linear recombination system in combination with the pSM3fr MCMV bacterial artificial chromosome in the *Escherichia coli* strain DY380 [49]. Sequence containing 4 repeated target sequences with complete complementarity to miR-142-3p were synthesized and inserted 86 bases into the 3’UTR of the IE3 gene (nucleotide coordinate—177898) of MCMV using linear recombination. Vector sequence containing the FRT flanked kanamycin cassette was inserted into the same region, with the kanamycin cassette removed from both viruses by FLP recombination. miR-142-3p target sequence with complementary sequences underlined: AGTCGACTCCATAAAGTAGGAAACACTACACGATTCCATAAAGTAGGAAACACTACAACCGGTTCCATAAAGTAGGAAACACTACACGATTCCATAAAGTAGGAAACACTACAACCGGT. The recombinant viruses have been checked for insertion by restriction analysis, Southern blotting, and sequencing. The targeted virus is referred to as MCMV-IE3-142 in this study while the control virus containing shuttle vector sequences but no miR-142-3p target sites is referred to as MCMV-IE3-015.

The MCMV-miR-142-3p vectors repaired for the MCK2 region (m131-129) were constructed using galactokinase- (galK-) mediated BAC recombination [101]. The SW105 E. coli strain carrying the MCMV Smith strain BAC with repaired MCK2 gene[50] was used to introduce an expression cassette encoding the galactokinase as well as the KanR gene flanked by 80bp homology arms to the targeted regions in the *ie3* 3’ untranslated region. Correctly recombined clones were identified based on the production of bright pink colonies on MacConkey agar containing kanamycin and Sanger sequencing of the inserted region. The galK-KanR cassette was replaced by homologous recombination with the miR-142-3p targeted insert (complementary sequences underlined as above): CGATGCACGGCTCCATAAAGTAGGAAACACTACATGCTAGCTGGCTCCATAAAGTAGGAAACACTACATGGACTGCGGCTCCATAAAGTAGGAAACACTACAGCATTGACGGCTCCATAAAGTAGGAAACACTACAGG. Correctly recombined clones were identified through negative selection on 2-deoxy-galactose-containing plates. These constructs were analyzed by restriction digest and Sanger sequencing of the inserted region before and after virus reconstitution. The targeted viruses is referred to as MCMV-IE3-142-rMCK2 in this study, while control virus lacking the miR-142-3p target sequences is referred to as MCMV-WT-rMCK2.

### Multi-step growth curves *in vitro*

3x10^5^ IC-21 macrophages or 2x10^5^ 3T3 or M2-10B4-fibroblasts were separately seeded into 6-well plates. One day later, cells were infected with either MCMV-IE3-142, MCMV-IE3-015, MCMV-IE3-142rMCK2, at a multiplicity of infection (moi) equalling 0.1 for 2 hours without centrifugal enhancement. After 2 hours, supernatant was collected for input virus titer (labelled day -1) and cells were washed with fresh media. Cells were scraped from duplicate wells immediately after the wash (day 0) and on days 1, 3, 5 and 7 after infection.

### Detection of GFP expression by flow cytometry

To visualize the regulation of gene expression by miR-142-3p, MCMV-GFP-142 and MCMV-GFP-015 were used to infect IC-21 macrophages or 3T3-fibroblasts respectively with MOI = 0.5, 3 or 10. Additionally, the regulation of gene expression by miR-142-3p was assessed on bone marrow derived macrophages and dendritic cells infected with MOI 5 either MCMV-GFP-142 or MCMV-GFP-015. The following day, cells were fixed and collected to determine GFP expression by flow cytometry.

### Cell depletions *in vivo*

In some experiments, CD4^+^ T cells, CD8^+^ T cells and/or NK cells were depleted 3 days before infection or 7 days after infection. Depletions were conducted by i.p. injection on days 3, 2 and 1 before infection or 7, 8 and 9 days after infection using 0.2 mg of anti-mouse CD4 mAb (clone GK1.5), anti-mouse CD8β mAb (clone 53–5.8) and/or anti-mouse NK1.1 mAb (clone PK136). Depletions were then maintained for the duration of the experiment by weekly injections with 0.15 mg (GK1.5) or 0.2mg (PK136 and 53–5.8) of antibody. All depleting antibodies were purchased from Bio-x-Cell. Depletions were confirmed by staining for CD4 (clone RM4-4), CD8α (clone 53–6.7) or NKp46 (clone 29A1.4).

### Virus titration

Nasal mucosa, lungs and salivary glands were collected at indicated time points post infection and frozen. Nasal mucosa was collected as previously described [51]. Twenty percent homogenates (w/v) were prepared from each collected tissue for virus quantification by plaque assay [51]. Briefly, tissues were weighed and homogenized using a pestle with a small amount of sterile sand in a 1.5 ml centrifuge tube, then suspended with RPMI supplemented with 10% FBS, 100 Units/mL penicillin, and 100 μg/mL streptomycin. Supernatants from the homogenate were collected after centrifugation (2400 xg, 10 min) and viral plaque assay was performed on M2-10B4 cells.

### Adoptive transfer of OT-I T cells

For adoptive transfer of OT-I T cells we used OT-I transgenic mice expressing CD45.1 as donor cells. Splenocytes containing 5000 OT-I cells from naïve transgenic mice were injected i.v. into sex-matched congenic recipients via the retro-orbital sinus suspended in 100 μl PBS. The following day, recipients were i.n. infected with 10^6^ PFU MCMV-Ova.

### Lymphocytes isolation, antibodies, tetramer staining, intracellular cytokine stimulation (ICS) and FACS analysis

Lymphocytes from the blood were collected from the retro-orbital sinus and mixed with 10 μl heparin (1000 units/ml). Lymphocytes from the spleen were collected by homogenization of the spleen through a 70 μM filter and suspended in T cell medium (RPMI-1640 medium with L-glutamine, 10% FBS, 100 units/ml Penicillin, 100 μg/ml Streptomycin, and 5 x 10^-5^M β-mercaptoethanol). CD4^+^ and CD8^+^ T cells were identified by antibodies specific for CD3 (clone 17A2), TCRβ (clone H57-597), CD4 (clone RM4-4) and CD8α (clone 53–6.7). OT-I donor cells were further distinguished from recipient cells by CD45.1 (clone A20) and TCR Vα2 (clone B20.1). MHC-I-tetramers loaded with peptides from M45 and M38 were generated at the NIH tetramer core facility (http://tetramer.yerkes.emory.edu/) and used to identify MCMV-specific CD8^+^ T cells as described previously [102]. For assessment of cytokine production after stimulation, splenocytes were stimulated with 1 μg/ml M38_316-323_ peptide (Genemed Synthesis, Inc), 1 μg/ml Brefeldin A (GolgiPlug, BD, Bioscience) in the presence of antibody specific for CD107a (clone 1D4B) at 37°C for 3 hours. Cells were chilled on ice, and live cells were discriminated with Zombie Aqua (Biolegend) prior to staining for expression of CD3 (clone 17A2), and CD8α (clone 53–6.7). Finally, splenocytes were fixed and permeabilized with Cytofix/Cytoperm (BD Biosciences), following the manufacturer’s instructions, and stained for intracellular TNF-α (clone MP6-XT22) and IFN-γ (clone XMG1.2). All antibodies were purchased from Biolegend and cells were collected on BD Fortessa and analyzed with FlowJo software (TreeStar). Gating strategies for flow analyses were shown in S4 Fig.

### DNA and RNA extraction and quantitative real-time PCR (qPCR)

For assessing viral genome copies, DNA was extracted from the salivary gland using 50 μl from a twenty percent tissue homogenate (w/v). For mandibular lymph nodes (manLNs), deep cervical lymph nodes (DCLNs) and mediastinal lymph nodes (MLNs), the whole lymph node was used after homogenization in RPMI-1640 medium using a pair of needles. In all cases, DNA was extracted using the Puregene core kit A (Qiagen) and following the manufacturer’s instructions for extraction of DNA from tissues. RNA was removed by adding RNase A solution and DNA was eluted with 30 μl distilled water. Two microliters DNA were used as a template in each qPCR reaction. The qPCR targeting MCMV-E1 gene was performed as previously described [93]. The genome copy numbers were calculated based on a standard curve of a plasmid containing the MCMV-E1 gene.

For assessment of viral RNA, bone marrow derived macrophages, bone marrow derived dendritic cells or M2-10B4 cells were infected with MOI 5 either MCMV-GFP-142 or MCMV-GFP-015 for 24 hours. RNA was extracted from infected cells using RNeasy Mini kit (Qiagen). DNA were removed using TURBO DNA-free Kit (Invitrogen). Afterwards, cDNA was produced using a high-capacity cDNA reverse transcription kit (Applied Biosystems). IE1 and IE3 transcripts were detected by individually primers and probes. For detecting IE1 gene expression, forward primer 5’-TCTGAGGAGCACCAGATACA-3’, reverse primer 5’-GTCCTCTCCACATAACTCATACAC-3’ and probe 5’-FAM-ACCATGCCTAGGATCAGAGGTGCTA-3’. For detecting IE3 gene expression, forward primer 5’-TCCTCCTCTCCTCAACACTT-3’, reverse primer 5’-CGCTCATCATCCTCATCATCTT-3’ and probe 5’- FAM-TTCCTCAGATCATGGTGGGCACAG-3’ (Integrated DNA Technologies). All samples were evaluated using a StepOnePlus system (Applied Biosystems). IE1 and IE3 gene transcripts were normalized to β-actin in each sample and evaluated using the ΔΔC_T_ method[103] and the formula: 2^-[(IE3_142_-β-actin_142_)-(IE3_015_-β-actin_015_)]. To assess expression of the miR-142-3p in different cell types, the TaqMan Advanced miRNA assay was used (ThermoFisher Scientific) according to the manufacturer’s instructions.

### Statistical analysis

Data in all experiments is pooled from at least two independent experiments. Error bars represent the standard error of the mean (SEM) unless specified otherwise in the figure legend. A two-tailed Student’s t test was used for statistical analysis for pairwise comparisons. All data analyses were performed in Graphpad Prism 6. For all statistical analysis, *p < 0.05, **p <0.01, ***p <0.005.

## Supporting information

S1 Fig The miR-142-3p is detected in bone marrow derived dendritic cells and macrophages, but not in M2-10B4 fibroblast cells. **A.** The miR-142-3p expression in different cell types was evaluated by TaqMan advanced miRNA assays (applied biosystems). Each symbol represents an individual sample. The solid line shows the mean value, and error bars represent the SEM. Data are from one representative experiment of two independent experiments. **B.** Shown are viral DNA copies in the salivary gland at 14 days post infection. **C.** Mice were infected with MCMV-IE3-142 via foot-pad inoculation. Viral titers were assessed in the salivary gland on day 14. Each symbol represents an individual animal. Control virus samples were derived from mice infected with either wild-type MCMV or MCMV-IE3-015.
(TIFF)

S2 Fig Confirmation of cell depletions. Representative FACS plots show the efficacy of CD4^+^ T cell depletion, CD8^+^ T cell depletion, CD4^+^/CD8^+^ T cell double depletion or NK cell depletion in the blood. Blood was collected before infection and every week during infection for flow analysis.
(TIFF)

S3 Fig No correlation between viral titers in the nasal mucosa and salivary gland. Data are from the animals shown in Fig 2A. C57BL/6 mice were depleted of NK cells (**A**) or CD4^+^and CD8^+^ T cells (**B**) beginning 3 days before i.n. infection with MCMV-IE3-142. Data were plotted to show the viral titers in the nasal mucosa vs. salivary gland for each mouse at day 14 post infection. Each symbol represents an individual animal and data are combined from two independent experiments.
(TIFF)

S4 Fig Viruses with repaired MCK-2 fail to spread efficiently in C57BL/6 mice. MCMV with a repaired MCK-2 gene was kindly provided by Dan Streblow. The miR-142-3p targeting sites were inserted into the 3’-UTR of the IE3 gene as described in the Experimental Methods section. **A.** Targeting IE3 with miR-142-3p binding sites prevents viral replication in miR-142-3p-expressing macrophages. Multi-step growth curves of the MCMV-WT-rMCK2 control virus and the MCMV-IE3-142-rMCK2 virus in M2-10B4 fibroblasts and IC-21 macrophages. **B and C.** Depletion of NK cells from C57BL/6 mice enables efficient dissemination of MCMV-IE3-142-rMCK2 from the nasal mucosa to the salivary gland. C57BL/6 mice were depleted of NK cells before i.n. infection with wild-type or miR-142-3p-targeted viruses with repaired MCK-2. Shown are the viral titers (**B**) and viral genome copies (**C**) in the salivary gland 14 days after infection. Each symbol represents an individual animal. The solid line shows the median value. Dashed line in **B** shows the plaque assay detection limit (50 PFU/g).
(TIFF)

S5 Fig Representative gating strategies. **A.** Representative gates show the basic gating strategy to identify live, CD8^+^ T cells. **B.** Representative MHC-I tetramer staining (data shown in Fig 5) after gating on CD8+ T cells as in **A**. **C.** Representative intracellular cytokine staining (ICS) of endogenous CD8^+^ T cells (gated as in **A**) after stimulation with the viral M38 peptide (data shown in Fig 5). **D.** Representative ICS of OT-I cells (gated first on CD8^+^ T cells as in **A**) after stimulation with the SIINFEKL peptide (data shown in Fig 5).
(TIFF)
