# Supplementary figures and images for: Hematopoietic cell-mediated dissemination of murine cytomegalovirus is regulated by NK cells and immune evasion
Source: PLoS Pathog. 2021 Jan 28;17(1):e1009255. doi: 10.1371/journal.ppat.1009255 (PMC7872266; doi:10.1371/journal.ppat.1009255)

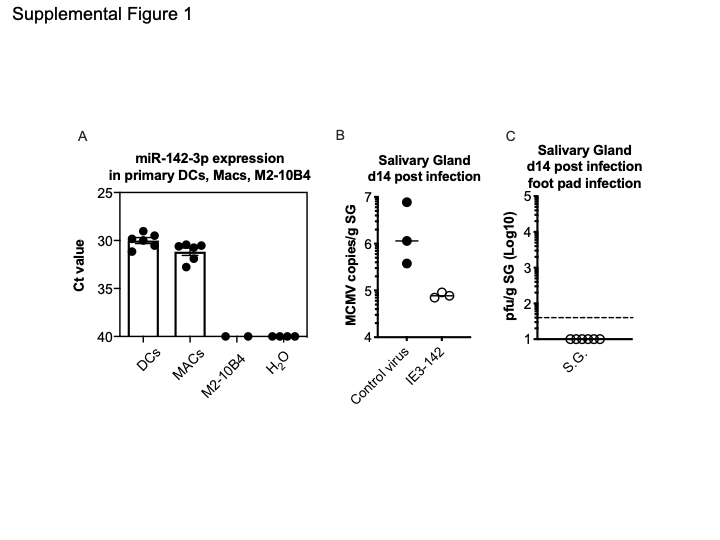

Supplement: S1 Fig — A. The miR-142-3p expression in different cell types was evaluated by TaqMan advanced miRNA assays (applied biosystems). Each symbol represents an individual sample. The solid line shows the mean value, and error bars represent the SEM. Data are from one representative experiment of two independent experiments. B. Shown are viral DNA copies in the salivary gland at 14 days post infection. C. Mice were infected with MCMV-IE3-142 via foot-pad inoculation. Viral titers were assessed in the salivary gland on day 14. Each symbol represents an individual animal. Control virus samples were derived from mice infected with either wild-type MCMV or MCMV-IE3-015. (TIFF) [file ppat.1009255.s001.tiff]

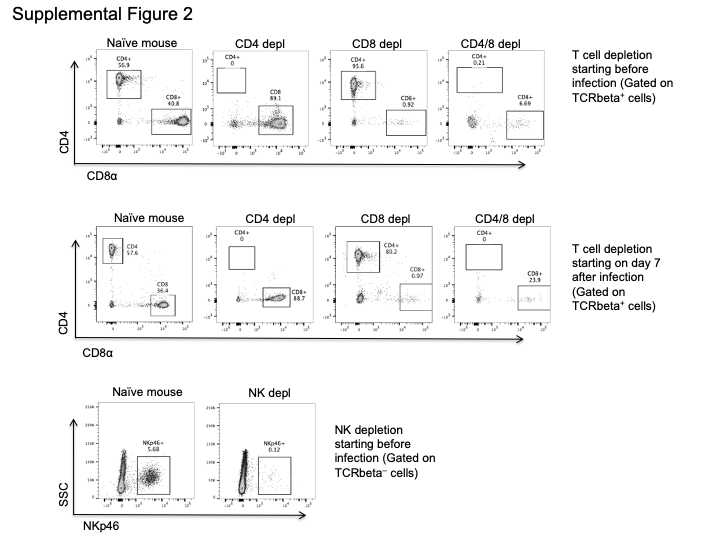

Supplement: S2 Fig — Representative FACS plots show the efficacy of CD4+ T cell depletion, CD8+ T cell depletion, CD4+/CD8+ T cell double depletion or NK cell depletion in the blood. Blood was collected before infection and every week during infection for flow analysis. (TIFF) [file ppat.1009255.s002.tiff]

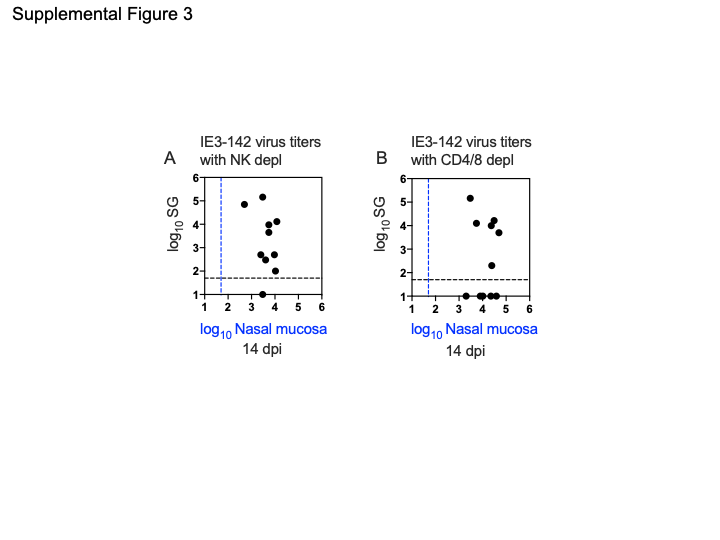

Supplement: S3 Fig — Data are from the animals shown in Fig 2A. C57BL/6 mice were depleted of NK cells (A) or CD4+and CD8+ T cells (B) beginning 3 days before i.n. infection with MCMV-IE3-142. Data were plotted to show the viral titers in the nasal mucosa vs. salivary gland for each mouse at day 14 post infection. Each symbol represents an individual animal and data are combined from two independent experiments. (TIFF) [file ppat.1009255.s003.tiff]

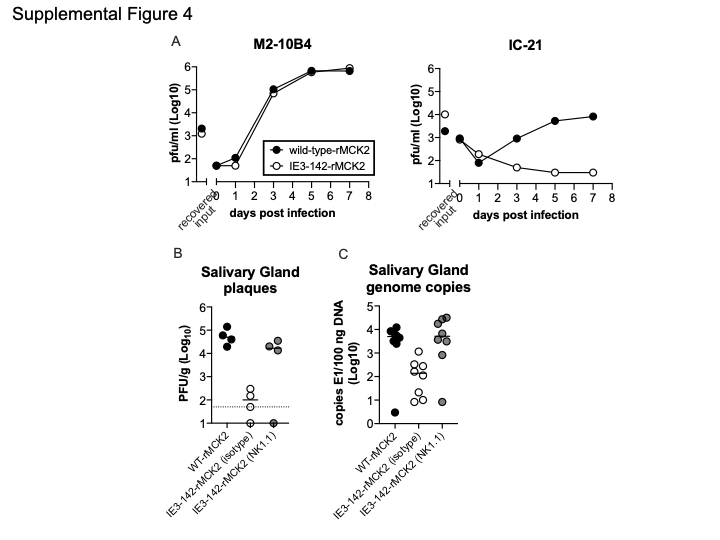

Supplement: S4 Fig — MCMV with a repaired MCK-2 gene was kindly provided by Dan Streblow. The miR-142-3p targeting sites were inserted into the 3’-UTR of the IE3 gene as described in the Experimental Methods section. A. Targeting IE3 with miR-142-3p binding sites prevents viral replication in miR-142-3p-expressing macrophages. Multi-step growth curves of the MCMV-WT-rMCK2 control virus and the MCMV-IE3-142-rMCK2 virus in M2-10B4 fibroblasts and IC-21 macrophages. B and C. Depletion of NK cells from C57BL/6 mice enables efficient dissemination of MCMV-IE3-142-rMCK2 from the nasal mucosa to the salivary gland. C57BL/6 mice were depleted of NK cells before i.n. infection with wild-type or miR-142-3p-targeted viruses with repaired MCK-2. Shown are the viral titers (B) and viral genome copies (C) in the salivary gland 14 days after infection. Each symbol represents an individual animal. The solid line shows the median value. Dashed line in B shows the plaque assay detection limit (50 PFU/g). (TIFF) [file ppat.1009255.s004.tiff]

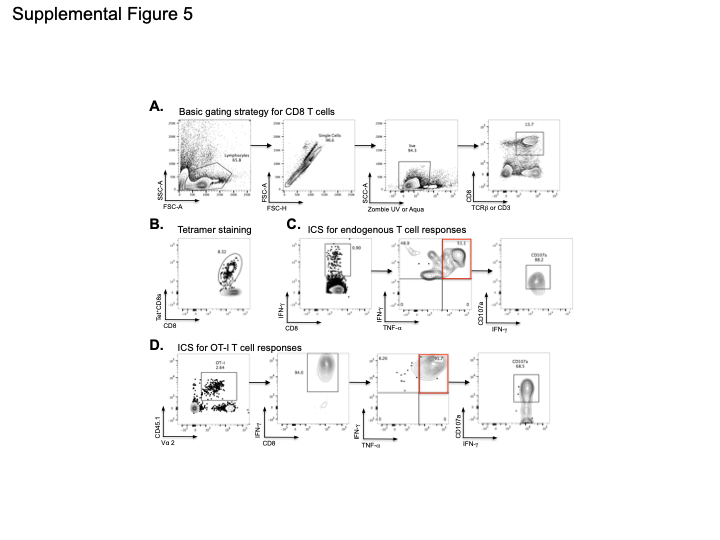

Supplement: S5 Fig — A. Representative gates show the basic gating strategy to identify live, CD8+ T cells. B. Representative MHC-I tetramer staining (data shown in Fig 5) after gating on CD8+ T cells as in A. C. Representative intracellular cytokine staining (ICS) of endogenous CD8+ T cells (gated as in A) after stimulation with the viral M38 peptide (data shown in Fig 5). D. Representative ICS of OT-I cells (gated first on CD8+ T cells as in A) after stimulation with the SIINFEKL peptide (data shown in Fig 5). (TIFF) [file ppat.1009255.s005.tiff]
